# Long-chain saturated free fatty acids induce pancreatic β−cell lipotoxic stress via H_2_O_2_−mediated mitochondria-associated membrane remodeling

**DOI:** 10.3389/fendo.2026.1852671

**Published:** 2026-07-23

**Authors:** Sarah Sharifi, Meike Knittel, Maren Böger, Anne Jörns, Ilir Mehmeti

**Affiliations:** Institute of Clinical Biochemistry, Hannover Medical School, Hannover, Germany

**Keywords:** ER-mitochondria coupling, H_2_O_2_, lipotoxicity, MAM formation, type 2 diabetes, β-cell failure

## Abstract

**Introduction:**

Type 2 diabetes (T2D) is characterized by progressive pancreatic β-cell dysfunction and loss, driven by chronic exposure to elevated circulating free fatty acids (FFAs). Mitochondria-associated endoplasmic reticulum membranes (MAMs), the specialized contact sites between the endoplasmic reticulum (ER) and mitochondria, regulate cellular metabolism and survival, yet their role in β−cell lipotoxicity remains unclear.

**Methods:**

We examined the impact of physiologically relevant long−chain saturated (palmitic and stearic acid) and unsaturated (palmitoleic and oleic acid) FFAs, applied alone or in combination, on MAM formation in insulin−secreting INS−1E cells. ER-mitochondria contacts were quantified by proximity−ligand assay (PLA), transmission electron microscopy and genetically encoded MAM sensors. The role of reactive oxygen species was investigated using the H_2_O_2_−scavenging enzyme glutathione peroxidase 8 (GPX8). To test causality, ER−mitochondria coupling was artificially enhanced with a synthetic ER−mitochondrial linker.

**Results:**

Saturated FFAs significantly increased ER-mitochondria contacts, whereas unsaturated FFAs had no negative effect and counteracted the saturated FFA-induced MAM formation. Expression of GPx8 suppressed palmitate−induced MAM formation, indicating that H_2_O_2_−mediated oxidative stress is required for the saturated FFA-induced MAM remodeling. Cells expressing the synthetic linker displayed heightened susceptibility to lipotoxic stress, confirming that sustained ER−mitochondria coupling promotes β−cell death.

**Conclusions:**

Our data demonstrate that dysregulated MAMs constitute a mechanistic link between saturated−FFA-induced lipotoxic stress and β−cell failure in T2D. Targeting MAM remodeling or the upstream H_2_O_2_ signal may represent a novel therapeutic avenue to preserve β−cell function under lipotoxic conditions.

## 1 Introduction

Modern lifestyle patterns, characterized by energy-dense, high-fat diets and physical inactivity, have increased the prevalence of obesity-related disorders, particularly type 2 diabetes (T2D) (1). Affecting more than 537 million people in 2021, T2D is a major global public health challenge (2). Once considered an adult-onset metabolic disease, its prevalence has risen substantially among children and adolescents (3), placing increasing burdens on families and health systems (2, 4). T2D arises from a complex interplay between insulin resistance in peripheral tissues and the progressive dysfunction of pancreatic β-cells (5). Both processes are strongly associated with elevated plasma levels of long-chain saturated free fatty acids (FFAs) (6, 7). This lipotoxicity is thought to be mediated through interconnected mechanisms, including oxidative and endoplasmic reticulum (ER) stress, with the ER-mitochondria axis representing a major site of these pathogenic events (8–11). Chronically increased levels of palmitic acid (PA), the most abundant saturated free fatty acid in plasma (7, 12), have been shown to enhance mitochondrial Ca^2+^ influx (10, 11, 13), leading to severe depolarization of the mitochondrial membrane potential and reduced ATP production in pancreatic β-cells. This effect involves the disruption of ER-Ca^2+^ homeostasis through redox-dependent inhibition of the sarco(endo)plasmic reticulum Ca^2+^-ATPase (SERCA) pump and most likely increased activity of Ca^2+^-releasing channels such as inositol 1,4,5-trisphosphate receptors (IP_3_Rs) (10, 11), highlighting a tight functional interplay between ER and mitochondria.

In fact, about 20% of the mitochondrial surface is closely juxtaposed with the ER, forming specialized contact regions termed mitochondria-associated membranes (MAMs) (14–17). MAMs are not merged but physically separated by flexible tethering complexes, maintaining a spacing of about 10–40 nm between both compartments (16, 18). These tethering complexes are composed of several proteins, notably the most recognized IP_3_Rs on the ER membrane and the voltage-dependent anion channel (VDAC) on the outer mitochondrial membrane, linked by the glucose-related protein 75 (GRP75) (19). This inter-organelle spacing is essential for MAMs to function as dynamic platforms that coordinate critical cellular processes, including energy metabolism, stress responses, and cell fate decisions (20–22). Deviations from the physiological spacing, in either direction, compromises MAM integrity, leading to cellular dysfunction and concomitant metabolic diseases, including T2DM (16, 23).

Evidence indicates that enhanced MAM formation contributes to glucotoxicity in pancreatic β-cells (24) and is further associated with insulin resistance and diabetes development (25, 26). In insulin-resistant and obese mouse models, a significant increase in MAM formation coincides with enhanced oxidative stress in the liver, ultimately leading to mitochondrial dysfunction and insulin resistance (25, 27). Likewise, elevated MAM formation has been implicated in renal dysfunction in diabetic kidney disease (28), diabetic cardiomyopathy (29), and in the alcohol-associated liver disease (30). On the other hand, a decreased proximity in MAMs has been associated with hepatic and skeletal muscle insulin resistance, with palmitate reducing ER-mitochondria interactions (31, 32). Controversially, recent studies point to the increased MAM formation in palmitate-mediated pancreatic β-cell death (33, 34). Thus, it remains unclear whether the increased or decreased proximity of MAMs is the primary driver of pancreatic β-cell lipotoxicity. Therefore, we set out to investigate the structure-activity relationships of saturated and unsaturated FFAs and their impact on MAM dynamics in insulin-secreting β-cells. Additionally, we assessed the role of cellular H_2_O_2_ in modulating MAM formation in these cells.

## 2 Materials and methods

### 2.1 Tissue culture of insulin-secreting INS-1E cells

Tissue culture of insulin-secreting INS-1E cells were cultured in RPMI 1640 medium (Thermo Fisher Scientific, Schwerte, Germany) supplemented with 10 mM glucose (Merck, Darmstadt, Germany), 10% (v/v) fetal calf serum (FCS; Thermo Fisher Scientific), 10 mM HEPES (SERVA, Heidelberg, Germany), 1 mM sodium pyruvate (Merck), 50 μM 2-mercaptoethanol (Merck), 10 U/mL penicillin (Santa Cruz Biotechnology, Santa Cruz, USA), and 10 μg/mL streptomycin (Merck) in a humidified atmosphere at 37 °C and 5% CO_2_, as described previously (35). INS-1E cells stably expressing glutathione peroxidase 8 in the ER (GPx8) have been generated and characterized as described before (9). The cells were routinely tested negative for mycoplasma contamination using the MycoAlert MycoPlasma Detection Kit (Lonza, Switzerland).

### 2.2 Preparation of free fatty acids and exposure of insulin-secreting INS-1E cells

Free fatty acids (FFAs) differing in chain length and degree of saturation - specifically palmitic acid (PA), stearic acid (SA), palmitoleic acid (POA), and oleic acid (OA) (all from Merck, Darmstadt, Germany) - were freshly dissolved in 90% ethanol at 60 °C. These FFAs were then applied at different concentrations in incubation media containing 1% FCS and FFA-free BSA (Serva, Heidelberg, Germany). The ratio between FFAs and added BSA was 0.5 mM: 1% (w/v) equivalent to a FFA/BSA molar ratio of 3.3 (11, 36). All control conditions contained amounts of BSA and ethanol equivalent to those present in the corresponding FFA treatment.

### 2.3 *In situ* proximity ligation assay

ER-mitochondria interactions were assessed by *in situ* proximity ligation assay (PLA). INS-1E cells were seeded at a density of 1 × 10^5^ cells per well on ECM-coated (Merck) 8-chamber slides (Sarstedt, Germany) and allowed to grow for 24 h. After treatment with FFAs and other compounds for the indicated time periods, PLA was performed using the NaveniFlex Cell MR Red kit (Navinci Diagnostics, Sweden) according to the manufacturer’s instructions. Briefly, cells were washed for 5 min in phosphate-buffered saline (PBS) and subsequently fixed with 4% paraformaldehyde at room temperature for 60 min or overnight at 4 °C. Thereafter, the cells were permeabilized with PBS containing 0.1% Triton X-100 for 20 min with gentle agitation. After thrice washing, the cells were blocked in kit-supplied blocking solution for 1 h in a humidity chamber at 37 °C then incubated with primary antibodies anti-VDAC1 (D73D12, 1:50) and anti IP_3_R1/3 (sc-3777518, 1:100) overnight at 4 °C. PLA was performed according to the manufaturing’s protocol. Finally, the cells were mounted with ProLong Glass Antifade Mountant with NucBlue nuclear stain (ThermoFisher) and imaged using an Olympus IX81 inverted microscope system equipped with an UPLSAPO 60 × and 1.35 numerical aperture super apochromatic silicon oil immersion objective. A constant z-step of 0.2 μm was used to acquire z-stacks of the whole cellular volume. Stacks were processed using CellSense software (Evident Olympus). For quantification, DAPI-positive nuclei and PLA puncta were separated into their respective fluorescence channels in CellSense and subsequently analyzed using a standardized image analysis workflow in Adobe Photoshop CS4 (Adobe Inc., USA) with identical threshold settings applied across all images. The total number of PLA puncta per image field was determined and normalized to the number of DAPI-stained nuclei within the corresponding image, as previously described (24, 37). For statistical analysis, biological replicates were considered independent observations in order to account for the experimental variability of the PLA assay.

### 2.4 Subcloning of SPLICS_S/L_-P2A-ER-mitochondria contact sensors and lentiviral transduction

For stable expression of split-GFP-based contact site sensors (SPLICS) in the insulin-secreting INS-1E cells, the cDNA of SPLICS_S_ Mt-ER Short P2A or SPLICS_L_ Mt-ER Long P2A (kindly provided by Marisa Brini & Tito Cali, Addgene plasmids # 164107 and # 164108; RRID: Addgene_164107; RRID: Addgene_164108), was specifically amplified with following composite primers (SPLICS_S/L_-fw 5’-ATACTAGTATGCGGGACCACATGGTGC-3’ and SPLICS_S/L_-rev 5’-ATAGGATCCTTATGTTCCTTTTTCATTTGGATCTTTG-3’). The PCR products were subsequently subcloned into the SpeI and BamHI restriction sites of the pLVX-EF1α-IRES-Puro (Clontech) for lentiviral transduction. Both constructs were verified by sequencing. For stable transduction of insulin-secreting INS-1E cells with the SPLICS constructs lentiviruses were prepared as described before in detail (38, 39). Briefly, 8 x 10^6^ 293T cells were transfected with the packaging plasmid pPAX2 (28.125 µg), the envelope plasmid pcDNA-MDG (9.375 µg), and the transfer plasmid LVX-EF1α-IRES-Puro_SPLICS (37.5 µg) by calcium phosphate precipitation. After 48 h the virus containing culture medium was collected and centrifuged for 5 min at 500 x g to remove detached cells and cell debris. Insulin-secreting INS-1E cells were infected with the viral supernatant. After 6 h of infection the viral supernatant was replaced by fresh medium. The cells were selected for SPLICS_S_ or SPLICS_L_ expression by puromycin (0.3 µg/ml) (InvivoGen, Toulouse, France).

### 2.5 Synthetic ER-mitochondrial linker

The insulin-secreting INS-1E cells were seeded at different densities depending on the further experimentation and allowed to attach for 24 h. Subsequently, the cells were transiently transfected with plasmids AKAP1-mRFP (control plasmid), AKAP1 (34–63)-mRFP-yUBC6 (OMM-ER linker, kindly provided by Professor György Hajnóczky, Thomas Jefferson University), using the jetOptimus transfection reagent, according to the manufacturer’s instructions (Polypus, Illkirch, France). After 48 h post-transfection, cells were treated with FFAs or the respective controls for the indicated experimental readouts.

### 2.6 Live-cell calcium imaging

Mitochondrial Ca^2+^ was monitored using the mitochondria-targeted, genetically encoded FRET Ca^2+^ sensor Cameleon 4mtD3cpv (kind gift from Amy Palmer and Roger Tsien; Addgene plasmid #36324; http://n2t.net/addgene:36324; RRID: Addgene_36324). 1 × 10^6^ INS-1E cells were electroporated with 3 μg of the Ca^2+^ sensor together with 3 μg of the OMM–ER linker plasmid or the corresponding control plasmid using the Neon Transfection System (Thermo Fisher Scientific) and cultured for 48 h before perifusion experiments. Continuous perifusion was performed as described elsewhere (10). Briefly, cells were perfused with a modified Krebs–Ringer (KR) solution containing (in mM): 134 NaCl, 4.8 KCl, 1.2 KH_2_PO_4_, 1.2 MgSO_4_, 25 HEPES, 3 glucose, and 1 CaCl_2_, supplemented with 1% (w/v) fatty acid–free BSA and the test compounds. Perfusion was maintained at 1 ml/min using a peristaltic pump (Ismatec, Zurich, Switzerland). Time−lapse images were acquired every 2 s on an Olympus IX81 inverted microscope equipped with a UPLSAPO 40×/1.35 NA objective and an incubation chamber maintaining 37 °C, 5% CO2, and 60% humidity (Olympus, Hamburg, Germany). Image acquisition and ratiometric analysis were performed in CellSens (Olympus). After background subtraction, individual cells were segmented (ROIs defined), and the fluorescence ratio for each cell was quantified over time.

### 2.7 Cell death detection

Activation of Caspase-3 was determined by flow cytometry using the CellEvent Caspase-3/7 Green or Red Detection Reagents (Thermo Fisher Scientific). 8 × 10^5^ insulin-secreting INS-1E cells were seeded onto 6-well plates and allowed to attach for 24 h before transient transfection with the corresponding plasmids (AKAP1-mRFP; OMM-ER linker or GFP, FATE-1-GFP (fetal and adult testis-expressed 1)). After 48 h of transfection, the cells were incubated with the indicated FFAs. Subsequently, the cells were trypsinized, stained, and washed according to the manufacturer’s protocol. The cells were suspended in 1 ml PBS and 10,000 positively transfected cells (RFP positive) from each sample were analyzed by flow cytometry (CyFlow ML, Partec, Münster, Germany) using the FL-1 channel (488 nm excitation/527 nm emission) and FL-10 channel (561 nm excitation/610 nm emission), while GFP-positive samples were analyzed on a CytoFLEX LX cytometer (Beckman Coulter, USA) using the B535-A and Y610-A detectors. Data were analyzed using FlowJo software (Tree Star, Ashland, OR, USA).

### 2.8 Electron microscopy

Mock- and GPx8-expressing insulin-secreting INS-1E cells were left untreated or treated with PA for 24 h before fixation in 2% paraformaldehyde and 2.5% glutaraldehyde in 0.1 M cacodylate buffer as described in detail (40). For ultrastructural analysis, thin sections of the cell pellets were viewed by an electron microscope (Morgagni 268, FEI, via Thermo Fisher Scientific, Frankfurt, Germany).

### 2.9 Reactive oxygen species and cell viability

Control and GPx8-expressing INS-1E cells were seeded onto 96-well plates (5 × 10^4^ cells/well). After 24 h, cells were loaded with 10 µM DCFDA-H_2_ for 40 min at 37 °C and subsequently left untreated or treated as indicated. DCF fluorescence was measured at 480/520 nm using a BioTek Synergy Mx fluorescence reader (Agilent Technologies). Cell viability was determined by the MTT assay (0.5 mg/mL MTT; Merck) using a BioTek PowerWave 340 microplate reader (Agilent Technologies). DCF fluorescence was normalized to cell viability and expressed relative to untreated controls.

### 2.10 Western blot analysis

Total cell lysates were sonified in phosphate-buffered saline (PBS; containing protease inhibitor mix (Roche Diagnostics, Manheim, Germany), and protein concentration was quantified by the BCA assay (Thermo Fisher Scientific). Equal amounts of protein (30 µg) were separated by 12.5% SDS-PAGE and transferred to a PVDF membrane (GE Healthcare Europe, Freiburg, Germany). After blocking with 5% non-fat dry milk (Carl Roth, Karlsruhe, Germany) for 1 h at RT, the membranes were incubated with anti-GFP antibody (#632375, Clontech, 1:15.000) overnight at 4 °C, followed by HRP-conjugated secondary antibodies (diluted 1:20,000, 1 h, RT) Chemiluminescence signal were visualised and acquired by using the ECL detection system (GE Healthcare Europe) and an automated chemiluminescence imager (INTAS Science imaging, Göttingen, Germany).

### 2.11 Statistical analysis

Unless stated otherwise, data are presented as means ± SEM. Graphs and statistical analyses were made with GraphPad Prism (GraphPad Software, San Diego,USA). Statistical significance was determined using one-way ANOVA prior to Dunnett’s or Bonferroi’s multiple comparison test and Student’s t-test, unpaired,two-tailed. P values <no><</no> 0.05 were considered statistically significant.

## 3 Results

### 3.1 Validation of the proximity ligation assay for detection of ER-mitochondria interaction

MAMs are increasingly recognized as important hubs involved in various cellular processes, including Ca^2+^ homeostasis, mitochondrial dynamics, and apoptosis (41). While disruption of MAMs has been consistently linked to insulin resistance in peripheral tissues (32, 42, 43), their role in beta cell toxicity under conditions of elevated FFAs remains unclear. To get insight into the role of MAMs in the lipotoxicity of pancreatic β-cells, we first set up the conditions to quantitatively assess ER-mitochondria contacts. Building on our previous findings that β-cell apoptosis features enhanced ER- to mitochondria Ca^2+^-transfer with concomitant ER and mitochondrial dysfunction (10, 11), we targeted the ER-resident IP_3_R1/3 and the mitochondrial outer-membrane channel VDAC1 to quantify functional ER-mitochondria coupling under lipotoxic conditions. To assess IP_3_R-VDAC1 proximity as a readout of MAM contacts in our cell model, we treated insulin-secreting INS-1E cells with 0.5 µg/ml tunicamycin, a pharmacological inhibitor of N-linked glycosylation commonly used to induce ER stress and reported to increase ER-mitochondria contacts (24, 25), for 2 or 6 h. As shown in **Figure 1A**, tunicamycin markedly increased the PLA signal, as indicated by increased punctate patterns of juxtaposed (≤ 40 nm) IP_3_R and VDAC proteins compared to vehicle-treated control cells, reflecting enhanced MAM contacts. PLA puncta were located within cell boundaries, as confirmed by DIC overlay images. Quantification of the PLA signals per nucleus revealed a significant increase in MAM contacts as early as 2 h after tunicamycin incubation, with a slightly stronger effect observed at 6 h (**Figure 1B**), reinforcing the reliability of the assay.

**Figure 1:**
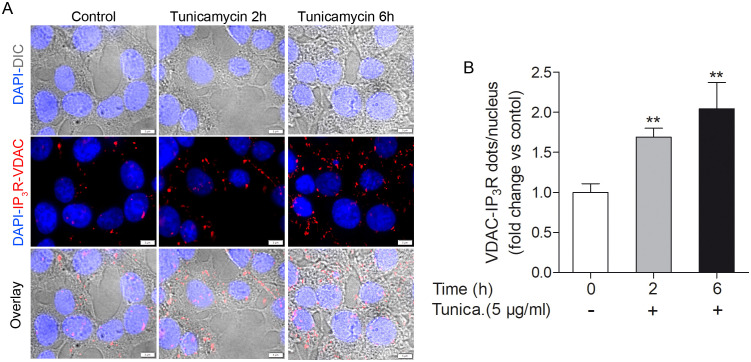
Validation of the *in-situ* proximity ligation assay (PLA) for detection of ER-mitochondria interaction. **(A)** Representative microscopy images showing the ER-mitochondria interactions as fluorescent red puncta resulting from IP_3_R–VDAC interactions. INS-1E cells were seeded 24 h prior incubation with 5 mg/ml tunicamycin for 2 or 6 h. Cells were then subjected to an *in-situ* proximity ligation assay, followed by counterstaining with DAPI (blue). **(B)** Quantification of red puncta per nucleus as a proxy of ER-mitochondria interaction. Data are means ± SEM of 5 independent experiments; **p<0.01 compared with cells incubated under control conditions (ANOVA/Dunnett’s test).

### 3.2 Time dependent ER-mitochondria interaction in response to long-chain saturated and mono-unsaturated fatty acids

Given that PA (~ 64 nmol/l) and SA (~ 22 nmol/l) are the most abundant long-chain saturated fatty acids in plasma (12) and exhibit pronounced cytotoxic effects in pancreatic β-cells (9, 11, 44), we investigated their impact on MAM dynamics in insulin-secreting INS-1E cells. Exposure of cells to both PA and SA resulted in enhanced MAM formation in a time-dependent manner (**Figures 2A, B**). In PA-treated cells, a significant increase in ER-mitochondria contact sites was already evident after 6 h and further increased after 24 h compared with vehicle-treated cells (**Figure 2A**). Similarly, SA treatment led to a significant rise in MAM formation at 6 h, followed by a slight reduction at 24 h (**Figure 2B**). In contrast, exposure of insulin-secreting cells to the corresponding unsaturated fatty acids POA or OA did not enhance ER-mitochondria proximity relative to control conditions (**Supplementary Figures 1A, B**). Thus, the potential of FFAs to increase the formation of MAMs in insulin-secreting β-cells seems to be limited to long-chain saturated fatty acids.

**Figure 2:**
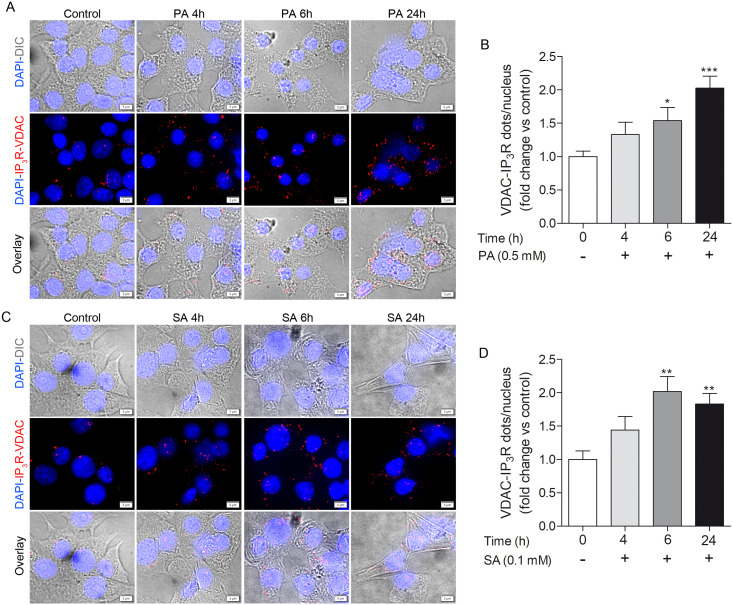
Time dependent ER-mitochondria interaction in response to long-chain saturated fatty acids. **(A, C)** Representative microscopy images showing the ER-mitochondria interactions as fluorescent red puncta resulting from IP_3_R-VDAC interactions. INS-1E cells were seeded 24 h prior incubation with 0.5 mM palmitic acid (PA) or 0.1 mM stearic acid (SA) for 4, 6 or 24 h. Cells were then subjected to an *in-situ* proximity ligation assay, followed by counterstaining with DAPI (blue). **(B, D)** Quantification of red puncta per nucleus as a proxy of ER-mitochondria interaction. Data are means ± SEM of 7 independent experiments; *p<0.05; **p<0.01; ***p<0.001 compared with cells incubated under control conditions (ANOVA/Dunnett’s test).

### 3.3 Impact of monounsaturated fatty acid co-treatment on saturated fatty acid-induced MAM formation and cytotoxicity

As the POA and OA did not affect MAMs in contrast to their saturated counterparts, we next examined whether addition of corresponding concentrations of POA or OA altered PA-induced MAM formation. Although PA still promoted an increase in MAM formation in the presence of POA, the extent of MAM formation decreased by 27% (Δ = 0.73) compared with PA alone (**Figure 3A**). Unlike POA, addition of equimolar concentrations of OA significantly abolished PA-mediated increase in MAM formation, resulting in 35% reduction (Δ = 65) in our insulin-secreting cell model (**Figure 3A**). Importantly, POA and OA also exerted a protective effect against SA (C18:0)-induced MAM formation; however, the attenuation of SA-mediated MAM formation was considerably less pronounced (19% POA, 26% OA) compared with the strong suppression observed in the PA condition (**Figure 3B**). Similar effects were observed for toxicity, such that co-incubation at equimolar concentrations was sufficient to abolish both PA- and SA-mediated toxicity, although the protective effect was weaker in the case of SA (**Figures 3C, D**). Compared with untreated cells, PA treatment yielded a residual cell viability of 60%, while co-treatment with POA or OA increased residual viability to 82 and 92%, respectively (**Figure 3C**). Likewise, both POA and OA counteracted the toxic effect of SA by 18 and 15%, respectively (**Figure 3D**). Thus, MAM formation appears to be closely associated with the toxicity profile of saturated FFAs, and unsaturated FFAs in particularly OA are capable of preserving MAM integrity in the presence of PA and, partially, in the presence of SA.

**Figure 3:**
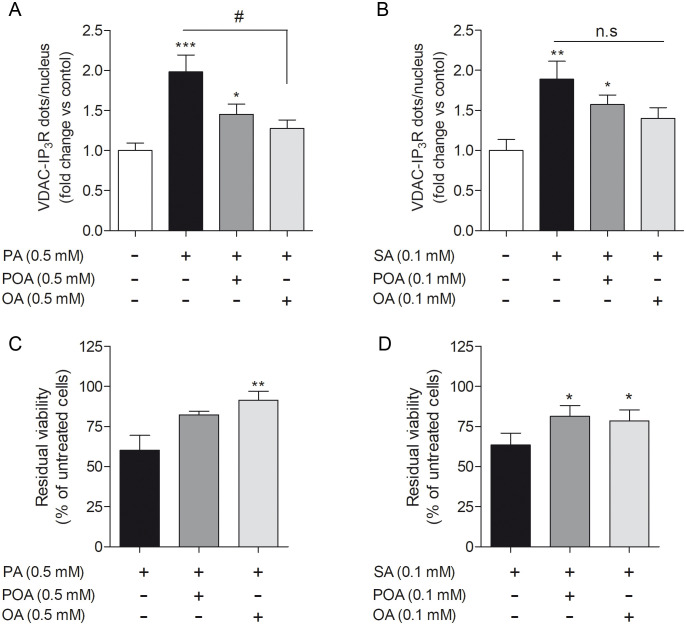
Impact of monounsaturated fatty acid co-treatment on saturated fatty acid-induced MAM formation and cytotoxicity. **(A, B)** Quantitative analysis of ER-mitochondria interactions. INS-1E cells were incubated under control conditions or treated for 24 h with **(A)** palmitic acid (PA, 0.5 mM) or **(B)** stearic acid (SA, 0.1 mM) alone or in combination with equivalent concentrations of palmitoleic acid (POA) and oleic acid (OA). IP3R-VDAC interactions were then assessed by *in-situ* proximity ligation assay. Data are means ± SEM of 4–6 independent experiments; *p<0.05; **p<0.01; ***p<0.001 compared with cells incubated under control conditions, ^#^p<0.05 compared to cells co-incubated with POA or OA (one-way ANOVA followed by Bonferroni’s multiple comparison test). **(C, D)** Cell viability was determined by MTT assay and expressed as % of vehicle-treated cells. Residual viability following 24 h treatment with PA alone or in combation with POA or OA **(C)** and with SA alone or in combination with POA and OA **(D)**. Data are means ± SEM of 6 independent experiments; *p<0.05; **p<0.01; compared with cells incubated with equimolar contractions of PA or SA (ANOVA/Dunnett’s test).

### 3.4 Impact of intracellular H_2_O_2_ on saturated fatty acid-induced MAM formation

Having established that unsaturated fatty acids are capable of abolishing PA and SA-induced MAM formation, we next sought to examine the potential mechanisms underlying this protective action. Previous studies have demonstrated that unsaturated fatty acids, particularly OA, protect β-cells from PA-mediated toxicity by attenuating both oxidative and ER stress, respectively (40, 45). Consistent with this notion, selective detoxification of H_2_O_2_ within the ER lumen by the H_2_O_2_-inactivating enzyme GPx8 has been shown to prevent PA-induced oxidative ER stress and subsequent lipoapoptosis in pancreatic β-cells (9–11). Therefore, we next assessed the potential involvement of intracellular H_2_O_2_ in mediating the effects of PA and SA on MAM formation. To confirm increased ROS production under our experimental conditions, intracellular ROS levels were measured using DCFDA-H_2_. Both PA and SA significantly increased ROS levels, whereas GPx8 markedly attenuated FFA-induced ROS formation (**Supplementary Figure 2**) We therefore next used β-cells stably expressing the ER-localized H_2_O_2_-detoxifying enzyme GPx8 (10) to examined MAMs under PA and SA treatment conditions. As shown in **Figure 4A**, PA still induced MAM formation in GPx8-expressing cells, but the induction was significantly attenuated, with GPx8 cells displaying 32% fewer ER-mitochondria contact sites than controls (Δ = 0.63) under identical PA conditions. In contrast, the protective effect of GPx8 against SA-mediated MAM formation was weaker (15%; Δ = 0.3; **Figure 4B**). Notably, expression of GPx8 itself had no adverse effect on MAMs under basal condition (**Figures 4A, B**). To further characterize the protective effect mediated by GPx8 at MAMs, we additionally performed ultrastructural analyses of control and GPx8-expressing cells (**Figure 4C**). In both control and GPx8-expressing cells, MAMs were detectable under control as well as lipotoxic conditions. As previously described (40), PA-treated control cells exhibited elongated ER cisternae, accompanied by increased ER-mitochondria interactions (indicated by arrows in **Figure 4C**). In contrast, GPx8-expressing cells displayed less pronounced ER cisternal elongation, together with a reduced number of MAMs compared with PA-treated control cells.

**Figure 4:**
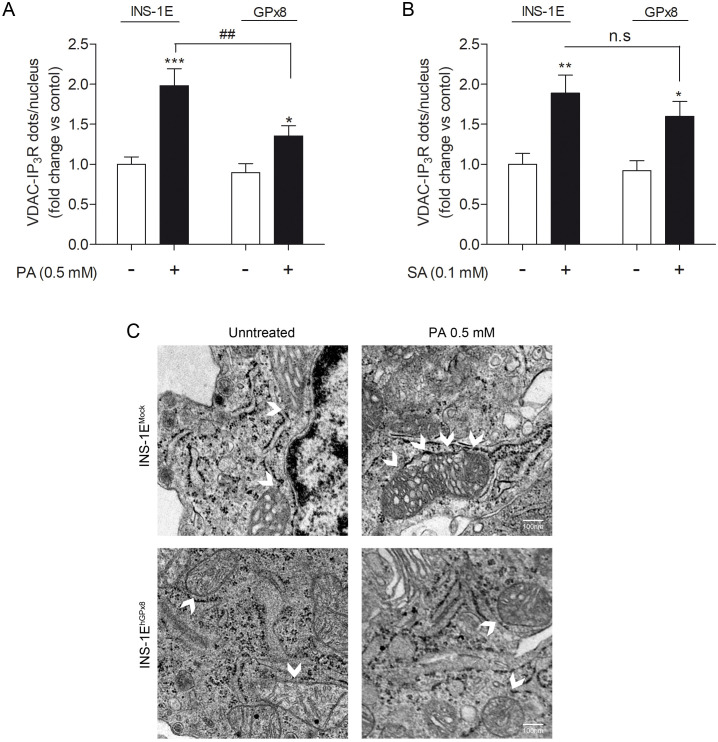
Impact of intracellular H_2_O_2_ on saturated fatty acid-induced MAM formation. **(A, B)** Quantitative analysis of ER-mitochondria interactions. Control and GPx8-expressing INS-1E cells were incubated under control conditions or treated for 24 h with **(A)** palmitic acid (PA, 0.5 mM) or **(B)** stearic acid (SA, 0.1 mM). IP3R-VDAC interactions were then assessed by *in-situ* proximity ligation assay. Data are means ± SEM of 4 independent experiments; *p<0.05; **p<0.01; ***p<0.001 compared with cells incubated under control conditions, ^##^p<0.01 compared with cells incubated under the same conditions (t-test, unpaired, two-tailed). **(C)** Representative transmission electron microscopic images from control and GPx8-expressing INS-1E cells under control and after 24 h incubation with PA. Arrows indicate ER membrane adjacent to mitochondria.

### 3.5 Impact of the synthetic MAM-linker on mitochondrial Ca^2+^ flux under lipotoxic conditions

Given that β-cell lipotoxicity is associated with increased Ca^2+^ transfer from the ER to mitochondria (10, 11), we investigated whether enhanced MAM formation contributes to altered mitochondrial Ca^2+^ flux in insulin-secreting INS-1E cells by expressing a synthetic outer mitochondrial membrane (OMM) and to the ER (OMM-ER linker). This construct is used to artificially increase the number of ER-mitochondria contact sites with a spacing of up to 5 nm (21, 46, 47). Consistent with this approach, PLA analysis revealed that MAM formation was significantly increased in pancreatic β-cells transfected with the OMM-ER linker compared with RFP-control plasmid-transfected cells (**Figure 5A**). Mitochondrial Ca^2+^ uptake was subsequently assessed exclusively in cells co-transfected with the OMM-ER linker and the mitochondrially-targeted Ca^2+^ sensor 4mtD3cpv (**Figure 5B**). As previously reported (10), perifusion of control cells and cells expressing the control construct with PA resulted in a marked increase in mitochondrial Ca^2+^ levels compared to basal perifusion (**Figure 5C**). In cells transfected with the OMM-ER linker, mitochondrial Ca^2+^ levels were significantly higher (**Figure 5D**), indicating that enhanced ER-mitochondria coupling mediated by the synthetic linker further increased Ca^2+^ flux into the mitochondria. This notion is further supported by the observation that Ca^2+^ uptake occurred at a faster rate in cells expressing the linker compared with control cells (0.50 ± 0.07 vs. 0.36 ± 0.08 min; **Figure 5E**). Thus, long-chain saturated FFAs potentiate ER-mitochondria Ca^2+^ transmission due to increased ER-mitochondria coupling.

**Figure 5:**
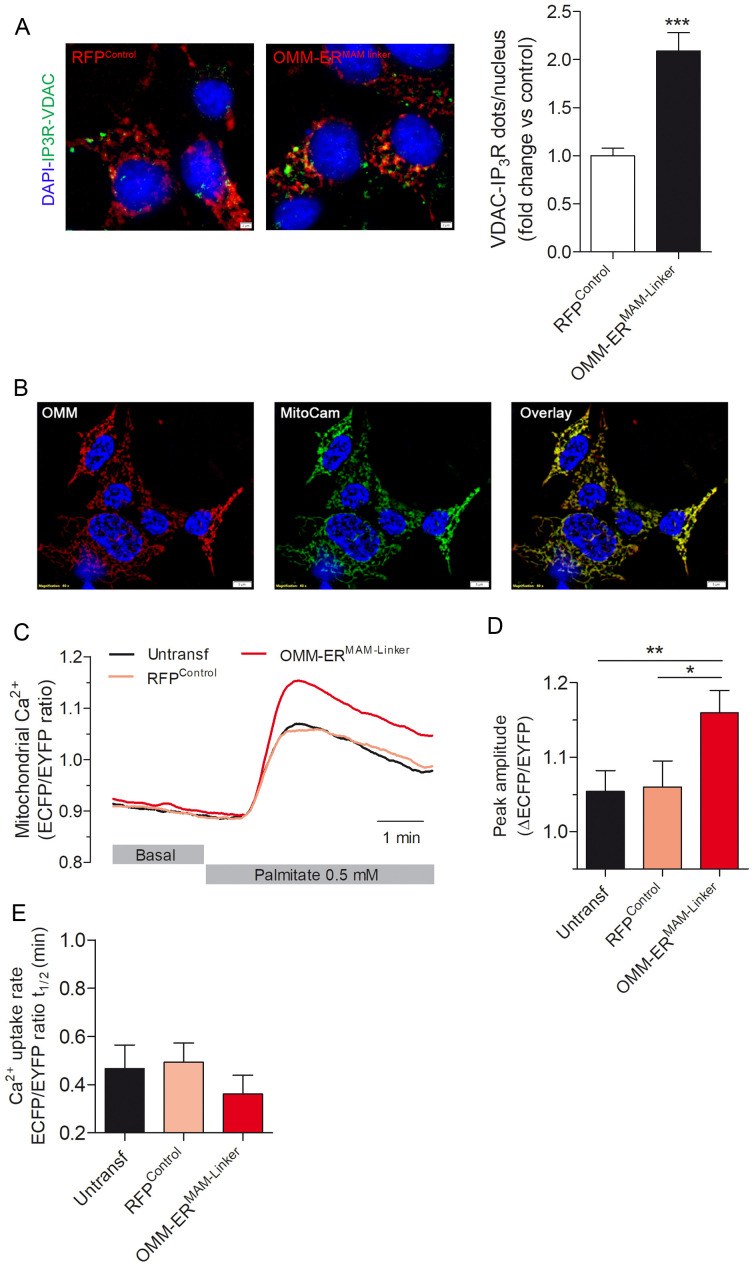
Impact of the synthetic MAM-linker on mitochondrial Ca^2+^ flux under lipotoxic conditions. **(A)** Effects of the ER-mitochondrial linker (OMM-ER^MAM-Linker^) on ER-mitochondria interactions. Representative microscopy images of INS-1E cells transfected with control plasmid (RFP^Control^) or the OMM-ER^MAM-Linker^ (red). IP_3_R-VDAC interactions were visualized as fluorescent green puncta assessed by *in-situ* proximity ligation assay, the corresponding quantitative analysis is depicted on the right. Data are means ± SEM of 3 independent experiments; ***p<0.001 compared with control cells (t-test, unpaired, two-tailed). **(B)** Immunofluorescent images of INS-1E cells co-expressing the OMM-ER^MAM-Linker^ (red) and the mitochondrially located calcium sensor (MitoCam), followed by counterstaining with DAPI. **(C)** Representative trace of mitochondrial Ca^2+^ dynamics upon perifusion under basal conditions (KR + 3 mM glucose + 1% FFA-free BSA), followed by perifusion with 0.5 mM PA-containing KR solution for 5 min. **(D)** Average peak amplitude of mitochondrial free Ca^2+^ in wild-type, RFP^Control^, and OMM-ER^MAM-Linker^-transfected cells in the presence of PA. **(E)** Half-time (t_1/2_) of mitochondrial Ca^2+^uptake in INS-1E control- and OMM-ER^MAM-Linker^ transfected cells during perifusion with PA. Data are means ± SEM of 4 independent experiments; *p<0.05; **p<0.01 compared with wild-type and RFP^Control^-transfected cells (t-test, unpaired, two-tailed).

### 3.6 Impact of synthetic MAM linker-mediated ER-mitochondria coupling on β-cell lipoapoptosis

Given the role of ER-mitochondria appositions in mediating ER to mitochondria Ca^2+^ transfer, we next investigated their causal involvement in β-cell lipoapoptosis. To do so, insulin-secreting INS-1E cells expressing either the corresponding control construct or the OMM-ER linker were exposed to the most abundant free fatty acid PA. As depicted in **Figure 6A**, incubation of control cells with PA resulted in a significant activation of caspase-3 in both control construct- and OMM-ER linker-expressing INS-1E cells compared with vehicle-treated cells. Importantly, cells transfected with the OMM-ER linker displayed a significantly increased susceptibility to PA-induced caspase-3 activation. In addition, under control conditions, ER-OMM linker-expressing cells exhibited a 23% increase in activated caspase-3 compared with cells expressing the control construct; however, this difference did not reach statistical significance. Controversially, expression of the ER-mitochondria spacer protein FATE1 significantly reduced the number of MAMs, as reflected by a decreased number of IP3R-VDAC PLA puncta compared with the corresponding control construct (**Supplementary Figures 3A, B**), consistent with previous reports describing FATE1 as an ER-mitochondria spacer that increases inter-organelle distance (42, 48). Under these conditions, PA-induced caspase-3 activation was not significantly altered compared with control-transfected cells and, in contrast to the ER-OMM linker, FATE1 expression did not further enhance β-cell apoptosis.

**Figure 6:**
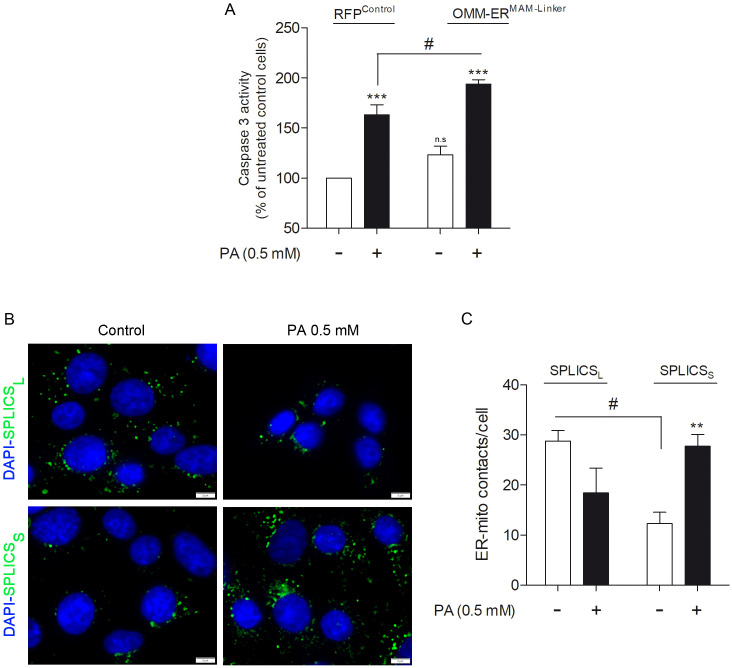
Impact of synthetic MAM linker-mediated ER-mitochondria coupling on β-cell lipoapoptosis. **(A)** INS-1E cells transfected with control plasmid (RFP^Control^) or the OMM-ER^MAM-Linker^ were incubated under control conditions or with palmitic acid (PA, 0.5 mM) for 24 h. Thereafter, the caspase-3 activity was determined by flow cytometry. Data are means ± SEM of 6 independent experiments; ***p<0.01, compared with control cells incubated under the same conditions; ^#^p<0.05 compared with cells under the same incubation condition (t-test, unpaired, two-tailed). **(B)** Representative fluorescence microscopy images of INS-1E cells expressing SPLICS_L_ and SPLICS_S_, showing fluorescent puncta upon excitation at 488 and 514 nm, indicative of GFP1–10 complementation (green), before and after incubation with 0.5 mM PA for 24 h. Nuclei were counterstained with DAPI (blue). **(C)** Quantification of SPLICS_L_ and SPLICS_S_ green puncta per cell. Data are means ± SEM of 3 independent experiments; **p<0.01 compared with cells incubated under control conditions, ^#^p<0.05 compared with cells incubated under the same conditions (t-test, unpaired, two-tailed).

To further validate the correlation between ER-mitochondria distance and lipotoxicity in pancreatic β-cells, we used two SPLICS sensors: SPLICS-Short, a split-GFP-based contact site sensor that reconstitutes bright GFP fluorescence at a distance of 8–10 nm, and SPLICS-Long, which detects contact sites at distances of 40–50 nm (49, 50). To this end, stable INS-1E cell lines expressing either SPLICS construct were generated to ensure homogeneous expression (**Supplementary Figure 4**). As shown in **Figures 6B, C**, INS-1E cells expressing either SPLICS-Short or SPLICS-Long revealed that, under control conditions, long-distance contacts were more frequent than short-distance contacts. However, upon exposure to PA for 24 h, the abundance of contact sites detected by SPLICS-Short dramatically increased compared to vehicle-treated cells, whereas the signal of SPLICS-Long decreased. This observation indicates a remodeling of ER-mitochondria contacts toward closer membrane apposition under lipotoxic conditions, which may contribute to enhanced ER-mitochondria Ca²^+^ transfer.

## 4 Discussion

Cellular organelles are no longer considered static and isolated entities, but rather dynamic and highly interconnected compartments. Accordingly, ER and mitochondria are physically linked through MAMs, which serve as dynamic platforms for interorganellar communication and are essential for maintaining cellular homeostasis (21, 22, 51). These platforms are increasingly recognized as essential sites in the regulation of fundamental cellular processes, including Ca^2+^ homeostasis, mitochondrial dynamics, stress responses, and apoptosis (41, 51). Accumulating body of evidence implicates MAM dysfunction in the pathogenesis of several diseases, including metabolic diseases such as diabetes mellitus (16, 23, 51).While disruption of MAM integrity has consistently been linked to insulin resistance in peripheral tissues (32, 42, 43, 52, 53), their role in β-cell lipotoxicity remains poorly understood.

In this study, we analyzed the effects of the physiologically most abundant saturated and unsaturated FFAs (12, 54), alone or in combination on MAM dynamics in insulin-secreting β-cells and evaluated the contribution of the ER-luminal H_2_O_2_ in this process. Additionally, artificially increased MAM formation and the ER-mitochondria spacing on Ca^2+^ homeostasis and apoptosis induction upon FFA exposure was analyzed. Long-chain saturated FFAs, PA and SA promoted MAM formation, whereas the unsaturated FFAs, POA and OA, did not adversely affect MAM integrity in our insulin-producing β-cell model. Instead, when applied in combination at equimolar concentrations, unsaturated FFAs, particularly OA, exerted a protective effect against PA- or SA-induced MAM formation. These observations are consistent with the known toxicity profiles of FFAs in pancreatic β-cells, where saturated FFAs induce pronounced cytotoxic effects (6, 11), whereas unsaturated FFAs such as OA are only marginally toxic and can mitigate PA-mediated β-cell toxicity (6, 40, 54, 55), pointing to a correlation between lipotoxicity and increased ER-mitochondria contacts in pancreatic β-cells. Accordingly, previous studies have reported that PA-induced apoptosis is accompanied by enhanced MAM formation in endocrine pancreatic β-cells (33, 34) and non-endocrine cells (56). Intriguingly, however, another study indicated that exposure of a mouse insulin-secreting cell line to lower concentrations of PA reduced ER-mitochondria interactions (52). Thus, MAM formation appears to be regulated in a concentration-dependent manner, with moderate PA levels triggering adaptive reductions in ER-mitochondria coupling, whereas supraphysiological PA concentrations, which are usually used to induce β-cell apoptosis under T2D conditions, are associated with increased contact formation between ER and mitochondria. This assumption is further supported by the induction of MAM-related tethering protein expression under identical incubation conditions (10, 33, 34). In line with this, several studies reported that pharmacological triggering of pro-apoptotic ER stress by tunicamycin coincides with elevated ER-mitochondria contacts (24, 25, 46).

Pancreatic β-cells possess limited endogenous antioxidant capacity due to low expression of H_2_O_2_-inactivating enzymes, rendering them particularly vulnerable to oxidative stress caused by H_2_O_2_ accumulation (9, 57, 58). Recent studies have demonstrated that β-cell lipotoxicity is closely linked to elevated H_2_O_2_ levels in the ER (9, 11, 59). An important source of H_2_O_2_ is the ER-oxidoreductin-1α, whose expression is significantly induced under lipotoxic conditions (11, 60). Elevated H_2_O_2_ levels within the ER lumen substantially contributed to impaired depletion of ER Ca^2+^ stores upon incubation with PA. The resulting Ca^2+^ release from the ER consequently led to increased Ca^2+^ transfer to mitochondria, causing mitochondrial Ca^2+^ overload and impairment of mitochondrial function. These findings suggest that H_2_O_2_ functionally modulates key Ca^2+^ transfer components such as IP_3_Rs and VDAC at MAMs (10, 11, 61), which together with the outer mitochondrial chaperone GRP75 serve as essential structural tethering proteins of MAMs (19, 21). Moreover, in addition to its classical localization in ER lumen, ERO1α is enriched at MAMs (62, 63), enabling it to directly influence MAM dynamics through local H_2_O_2_ production (64). The present study indeed demonstrates that eliminating H_2_O_2_ within the ER significantly attenuates the increase in MAM formation induced by saturated free fatty acid PA. This indicates that H_2_O_2_ not only regulates Ca^2+^ signaling between the ER and mitochondria, but also plays a role in regulating the ER-mitochondria apposition, thereby substantially contributing to β-cell dysfunction under lipotoxic conditions.

In agreement with this, the functional relevance of increased ER-mitochondria contact sites for lipotoxicity in pancreatic β-cells was further supported by the use of a synthetic ER-mitochondrial linker (46, 47). Cells with artificially enhanced ER-mitochondria contacts exhibited a markedly increased sensitivity towards toxicity induced by PA, underscoring that excessive and sustained ER-mitochondria coupling plays a key role in exacerbating β-cell death, supporting recent reports (33, 34). Importantly, while lipotoxic stress promotes a remodeling of ER-mitochondria contact sites toward closer membrane apposition, accompanied by increased mitochondrial Ca²^+^ accumulation and β-cell apoptosis, the same structural entity can mediate adaptive and protective responses under GLP-1 receptor agonist signaling (64). In this context, GLP-1 receptor signaling has been shown to organize ER-mitochondria contacts via VAPB and SPHKAP into a regulated signaling hub that promotes localized cAMP/PKA signaling, mitochondrial remodeling, and β-cell survival (65). Together, these observations suggest that the biological outcome of ER-mitochondria contact site formation depends not only on the extent of organelle coupling but also on their molecular composition and spatial arrangement.

In conclusion, to our knowledge, this study provides the first comparative analysis of the effects of physiologically relevant long-chain saturated and unsaturated FFAs, alone and in combination, on MAM dynamics and defines whether increased or reduced ER- mitochondria contact formation is associated with lipotoxicity in insulin-secreting β-cells. β-cell toxic saturated FFAs, including PA and SA induced a pronounced increase in MAM formation, whereas the unsaturated FFAs such as POA and OA had little impact on MAM integrity. Notably, unsaturated FFAs, particularly OA, efficiently counteracted enhanced MAM formation caused by saturated FFAs. Moreover, the ability of the H_2_O_2_-inactivating enzyme GPx8 to attenuate PA-driven enhancement of ER-mitochondria contacts suggests a role for H_2_O_2_ in mediating saturated FFA-induced changes in MAM integrity. Finally, our data provide causal evidence that chronically tightened ER-mitochondria contacts increases β-cell susceptibility to FFA-mediated toxicity. Using an ER-mitochondrial linker as an independent experimental approach, we demonstrate that excessive and sustained ER-mitochondria contacts exacerbate β-cell death in response to saturated FFAs. Collectively, these findings support the concept that a tightly regulated balance of ER-mitochondria coupling is critical for β-cell survival and that disruption of this balance toward enhanced ER-mitochondria contacts may contribute to β-cell lipoapoptosis. Further studies are warranted to determine whether similar ER-mitochondria remodeling occurs in primary pancreatic islets under lipotoxic conditions.

## Data Availability

Data are available in the article/**Supplementary Material** itself.
