# Supplementary material for: Long-chain saturated free fatty acids induce pancreatic β−cell lipotoxic stress via H2O2−mediated mitochondria-associated membrane remodeling
Source: Front Endocrinol (Lausanne). 2026 Jul 23;17:1852671. doi: 10.3389/fendo.2026.1852671 (PMC13441816; doi:10.3389/fendo.2026.1852671)
Supplement: Supplementary file 1 [file SupplementaryFile1.docx]

**Supplemental information**

Long-chain saturated free fatty acids induce pancreatic β‑cell lipotoxic stress via H_2_O_2_‑mediated mitochondria-associated membrane remodelling

**Sarah Sharifi,** **Meike Knittel, Maren Böger, Anne Jörns, and Ilir Mehmeti^*^**

Institute of Clinical Biochemistry, Hannover Medical School, 30625 Hannover, Germany

**^*^**Corresponding author:

PD Dr. Ilir Mehmeti, Institute of Clinical Biochemistry, Hannover Medical School, 30625 Hannover, Germany

E-mail: mehmeti.ilir@mh-hannover.de

Phone: +49 511 5326329

**Suppl. Fig. 1. Impact of unsaturated fatty on MAM formation**. Representative PLA images and quantitative analysis of ER-mitochondria interactions. INS-1E cells were incubated under control conditions or treated for 24 h with 0.5 mM palmitoleic acid (POA) or oleic acid (OA). IP3R-VDAC interactions were then assessed by in-situ proximity ligation assay. Data are means ± SEM of 4 independent experiments.

**Suppl. Fig. 2. Impact of GPx8 expression on PA- and SA-induced ROS formation.**  Intracellular ROS levels were determined using the fluorescent probe DCFDA-H_2_. Cells were left untreated or treated with PA (0.5 mM) or SA (0.1 mM) for 24 h. DCF fluorescence was normalized to cell viability determined by the MTT assay. Data are means±SEM of 4 independent experiments.

**Suppl. Fig. 3. Impact of MAM protein spacer on ER-mitochondria coupling and β-cell lipoapoptosis. (**A) Effects of the ER-mitochondrial spacer (FATE1^MAM-Spacer^) on ER-mitochondria interactions. Representative microscopy images of INS-1E cells transfected with control plasmid (GFP^Control^) or the FATE1^MAM-Spacer^ (green). IP3R-VDAC interactions were visualized as fluorescent red puncta assessed by in-situ proximity ligation assay, the corresponding quantitative analysis is depicted on the right. Data are means ± SEM of 3 independent experiments; *p<0.05 compared with control cells (t-test, unpaired, two-tailed). (B) INS-1E cells transfected with GFP^Control^ or the FATE1^MAM-Spacer^ were incubated under control conditions or with palmitic acid (PA, 0.5 mM) for 24 h. Thereafter, the caspase-3 activity was determined by flow cytometry. Data are means ± SEM of 4 independent experiments; ***p<0.01, compared with control cells (t-test, unpaired, two-tailed).

**Suppl. Fig. 4. Stable expression of SPLICS short/long and their subcellular localization in INS-1E cells.** (A) Representative immunoblot showing expression of SPLICS-Short (SPLICS-S) and SPLICS-Long (SPLICS-L) in mock-transfected (Mock) and stably expressing INS-1E cells. β-Actin served as a loading control. (B) Representative fluorescence images showing the subcellular localization of SPLICS-Short and SPLICS-Long in INS-1E cells. Mitochondria were stained with 250 nM MitoTracker Deep Red (ThermoFisher) for 30 min. Images were acquired using an UPLSAPO 60×/1.35 NA oil-immersion objective (Olympus).
